# Defective FGFR1 Signaling Disrupts Glucose Regulation: Evidence From Humans With *FGFR1* Mutations

**DOI:** 10.1210/jendso/bvae118

**Published:** 2024-06-13

**Authors:** Maria I Stamou, Crystal J Chiu, Shreya V Jadhav, Vanessa Ferreira Lopes, Kathryn B Salnikov, Lacey Plummer, Margaret F Lippincott, Hang Lee, Stephanie B Seminara, Ravikumar Balasubramanian

**Affiliations:** Reproductive Endocrine Unit and Harvard Center for Reproductive Medicine, Massachusetts General Hospital, Harvard Medical School, Boston, MA 02114, USA; Reproductive Endocrine Unit and Harvard Center for Reproductive Medicine, Massachusetts General Hospital, Harvard Medical School, Boston, MA 02114, USA; Reproductive Endocrine Unit and Harvard Center for Reproductive Medicine, Massachusetts General Hospital, Harvard Medical School, Boston, MA 02114, USA; Reproductive Endocrine Unit and Harvard Center for Reproductive Medicine, Massachusetts General Hospital, Harvard Medical School, Boston, MA 02114, USA; Reproductive Endocrine Unit and Harvard Center for Reproductive Medicine, Massachusetts General Hospital, Harvard Medical School, Boston, MA 02114, USA; Reproductive Endocrine Unit and Harvard Center for Reproductive Medicine, Massachusetts General Hospital, Harvard Medical School, Boston, MA 02114, USA; Reproductive Endocrine Unit and Harvard Center for Reproductive Medicine, Massachusetts General Hospital, Harvard Medical School, Boston, MA 02114, USA; MGH Biostatistics Center and MGH Division of Clinical Research (DCR) Biostatistics Unit, Massachusetts General Hospital, Harvard Medical School, Boston, MA 02114, USA; Reproductive Endocrine Unit and Harvard Center for Reproductive Medicine, Massachusetts General Hospital, Harvard Medical School, Boston, MA 02114, USA; Reproductive Endocrine Unit and Harvard Center for Reproductive Medicine, Massachusetts General Hospital, Harvard Medical School, Boston, MA 02114, USA

**Keywords:** FGFR1, diabetes, insulin resistance, genetics, human mutations

## Abstract

**Context:**

Activation of fibroblast growth factor receptor 1 (FGFR1) signaling improves the metabolic health of animals and humans, while inactivation leads to diabetes in mice. Direct human genetic evidence for the role of FGFR1 signaling in human metabolic health has not been fully established.

**Objective:**

We hypothesized that individuals with naturally occurring *FGFR1* variants (“experiments of nature”) will display glucose dysregulation.

**Methods:**

Participants with rare *FGFR1* variants and noncarrier controls. Using a recall-by-genotype approach, we examined the β-cell function and insulin sensitivity of 9 individuals with rare *FGFR1* deleterious variants compared to 27 noncarrier controls, during a frequently sampled intravenous glucose tolerance test at the Reproductive Endocrine Unit and the Harvard Center for Reproductive Medicine, Massachusetts General Hospital. *FGFR1*-mutation carriers displayed higher β-cell function in the face of lower insulin sensitivity compared to controls.

**Conclusion:**

These findings suggest that impaired FGFR1 signaling may contribute to an early insulin resistance phase of diabetes pathogenesis and support the candidacy of the FGFR1 signaling pathway as a therapeutic target for improving the human metabolic health.

Reproduction and metabolism have been known to be intricately linked for decades, yet the underlying precise pathomechanisms that define this intersection remain to be fully elucidated. One potential pathway that could merge those two domains is the fibroblast growth factor (FGF) signaling pathway, which has been implicated in developmental patterning and cell proliferation of many organs, including the hypothalamic neuroendocrine system and the pancreas. Attenuated signaling through the fibroblast growth factor receptor 1 (FGFR1) leads to diabetes in mice [1]. In addition, activation of FGFR1 signaling has been shown to improve the metabolic health of rodents, nonhuman primates, and humans [2-4]. Common and rare *FGFR1* variants have been implicated in metabolic traits, including waist-to-hip ratio, body mass index (BMI), obesity, type 2 diabetes (T2D), and fasting glucose levels in population-based genetic epidemiology studies [5-10]. These observations strongly implicate FGFR1 signaling as an essential determinant of glucose homeostasis in humans.

Rare naturally occurring *FGFR1* mutations contribute to approximately 12% of the genetic etiology of isolated hypogonadotropic hypogonadism (IHH) with or without anosmia, a rare inherited disorder of infertility that is caused by deficiency in the hypothalamic secretion of the gonadotropin-releasing hormone (GnRH) [11]. The mechanism by which *FGFR1* mutations cause IHH is haploinsufficiency (heterozygous inactivating/missense alleles). These “experiments of nature” mutations provide a framework to study the pleiotropic effects of haploinsufficient loss of function (LoF) of *FGFR1*, and patients harboring such mutations offer a unique opportunity to directly test the affect of altered FGFR1 signaling on glucose metabolism. In this study, we hypothesized that humans with rare LoF single-nucleotide variations (SNVs) in *FGFR1* will display defects in β-cell function and insulin action. To test this hypothesis, we examined the β-cell function and insulin sensitivity in IHH individuals with rare deleterious *FGFR1* SNVs compared to healthy noncarrier controls during a frequently sampled intravenous glucose tolerance test (FSIGT). In addition, to corroborate the findings from the rare variant studies, we examined the role of common *FGFR1* variants in the metabolic health of participants of the large hospital-wide cohort of the Massachusetts General Brigham Biobank (MGBB).

## Material and Methods

### Study Approval

This research was reviewed and approved by the Massachusetts General Brigham Institutional Review Board (IRB protocol 2009P002349). All participants provided written informed consent. Participants were recruited from 2 recallable genetics studies. *FGFR1* mutation carriers were participants with IHH in a longstanding and ongoing genetics study at Massachusetts General Hospital (MGH), Reproductive Endocrine Unit (IRB protocols 1999P006955 and 2020P000762), which has been reviewed and approved by the MGH Partners IRB. Noncarrier controls were participants in the MGBB, which has been reviewed and approved by the MGB IRB (protocol 2009P002312). Common variant association analysis was conducted as secondary use of clinical/research data under the MGH IRB protocol 2020P000762, which has been reviewed and approved by the MGB Institutional Review Board.

### Case Selection and Recruitment of the *FGFR1*m Cohort From Massachusetts General Hospital Reproductive Endocrine Unit

Using a recall-by-genotype approach, we searched whole-exome sequencing (WES) data within the MGH Reproductive Endocrine Unit cohort (n = 1394) for individuals with confirmed IHH and rare SNVs in *FGFR1*—NM 0231103 (*FGFR1* mutation carriers—*FGFR1*m cases). WES was performed as previously described [11]. Rare SNVs were defined by a minor allele frequency of less than 0.1% in the control database—gnomAD [12]. Among the 1394 IHH participants with WES data available, 175 IHH probands were found to carry SNVs in *FGFR1* (58 probands harbored protein-truncating variants [PTVs] and 117 probands harbored missense SNVs, Supplementary Table 1 [13], ClinVar accession Nos. SCV003932463 and SCV0039332605). Individuals with IHH were prioritized for recruitment if they harbored rare SNVs in *FGFR1* that disrupt *FGFR1-c* isoform that were (1) predicted PTVs (ie, nonsense, frameshift, essential splice site) or, also recruited, (2) missense variants that were predicted to be deleterious by in silico and in vitro analyses . In silico analysis included the utilization of the following prediction programs: CADD, Polyphen 2, SIFT, Mutation Taster, MutPred Score, REVEL, and Eve (Supplementary Table 2 [13]). Among those probands, 9 participants (7 men and 2 women) with heterozygous rare SNVs in *FGFR1* were enrolled and all participated in the study visits. Seven participants carried PTVs: c.2038C > T p.(Gln680*), c.1864C > T p.(Arg622*), c.154C > T p.(Gln52*), c.1039dup p.(Ile347Asnfs*61), c.1727_1734del p.(Arg576Profs*77), c.925C > T p.(Gln309*), c.1553-2A > G, and 2 participants carried missense variants: c.289G > A p.(Gly97Ser) and c.2102A > G p.(Tyr701Cys), with the majority being predicted to be pathogenic and likely pathogenic based on the American College of Medical Genetics criteria and absent from the control cohort of gnomAD (see Supplementary Table 2 [13]). All variants had a CADD score greater than 20, and missense SNVs were predicted to be deleterious based on in silico analyses (see Supplementary Table 2 [13]). The *FGFR1* SNVs were the primary genetic cause for IHH in the enrolled participants. The participants with rare *FGFR1* SNVs were diagnosed with either normosmic IHH or IHH with anomia (Supplementary Table 3 [13]), that was defined based on formal smell testing using the University of Pennsylvania Smell Identification Test (UPSIT) [14] or self-reported complete inability to smell and were all supplemented with sex steroids at the time of the study. *FGFR1* rare SNV carriers demonstrated additional nonreproductive phenotypes that have previously been linked to mutations in the IHH-related FGF-pathway genes, including hearing loss, bone abnormalities, teeth defects, and eye disorders (see Supplementary Table 3 [13]).

### Control Selection and Recruitment of *FGFR1*c Cohort From the Massachusetts General Brigham Biobank

The MGBB cohort (n = 65 247) was used to identify participants with no *FGFR1* mutations (noncarrier controls—*FGFR1*c). For the recruitment, a recontact letter was sent from the biobank staff along with an invitation letter, cosigned by the Biobank principal investigator and the principal investigator of this study, and an opt-in or opt-out letter to patients. Ten business days after the biobank sent the recontact letters to patients, each patient was directed to be contacted for recruitment, and a list of the response status for each patient was sent back to the biobank. Among the 808 MGBB participants who were contacted by our study team, 45 were enrolled in the study. Sixteen were excluded from participation after failing to meet one or more of the inclusion criteria for the study (discussed later). Twenty-seven healthy controls (16 men and 11 women) were enrolled. WES data of the MGBB participants showed no SNVs in *FGFR1*.

### Inclusion Criteria for the Entire Study Cohort

Inclusion criteria included the following: (i) no history of bleeding or thromboembolic disorders (ie, thrombocytopenia, deep vein thrombosis, pulmonary embolism, cerebrovascular disease, warfarin treatment, hypercoagulability syndromes); (ii) no history of illicit drug or heavy alcohol use (>4 g of alcohol per day); (iii) stable weight for previous 3 months; (iv) not currently pregnant; all female participants were administered a urinary pregnancy test to rule out pregnancies; (v) serum hemoglobin greater than or equal to 10 g/dL and baseline hematocrit greater than or equal to 38%; (vi) no current or previous diagnosis of type 1 or T2D as defined by the American Diabetes Association criteria: fasting glucose greater than 126 mg/dL or random blood glucose greater than 200 mg/dL on 2 occasions; and (vii) not currently taking medications that may influence glucose metabolism (eg, corticosteroids, thiazide diuretics).

### Age, Sex, Gender, and Ethnicity Background of the Enrolled Participants

The age, sex, and gender of the enrolled participants is provided in Supplementary Table 3 [13]. The ethnicity and race background of the enrolled participants was as follows: In the *FGFR1*m group, 5 participants identified as not-Hispanic White, 2 as Asian, 1 as Black, and 1 as having more than 1 ethnicity background, while the ethnicity of the enrolled *FGFR1*c cohort was noted as follows: not-Hispanic White (n = 20), Hispanic White (n = 2), Hispanic Unknown (n = 2), Asian (N = 2), and Black (N = 1).

### Study Visits

(A) Screening visit: Participants were evaluated with (i) collection of a detailed medical history; (ii) physical examination; (iii) collection of a blood sample for a detailed laboratory evaluation: complete blood count, complete metabolic panel, lipids, C-reactive protein, sex steroid hormones, glycated hemoglobin A_1c_ (HbA_1c_), pituitary gonadotropins, prolactin, and thyrotropin.
(B) Study visit: Prior to the study visit, the participants were fasted overnight for 8 hours. The following procedures were conducted during the study visit:
  - Anthropometric measurements were collected, including body weight, height, and body mass index (BMI) using simple measurement tools.
  - An FSIGT was performed to evaluate glucose metabolism in the study participants. The FSIGT is a widely accepted, well-established, and frequently used research procedure to assess glucose metabolism and β-cell function in humans. FSIGT provides estimates of insulin sensitivity that correlate significantly with those from the hyperinsulinemic euglycemic clamp [15] and is less complex compared to glucose clamp studies. Even though an oral glucose tolerance test could be used as an alternative approach, the FSIGT was chosen to help define precise effects during the first and second phases of insulin secretion and estimate both glucose effectiveness and the disposition index (D_I_) [16]. The FSIGT began with baseline sampling for plasma insulin and glucose at −10 and −1 minutes. These measurements were followed by the administration of a 0.3 g/kg bolus of glucose within 2 minutes. Subsequent venous blood samples were obtained at 1, 2, 3, 4, 5, 6, 8, 10, 12, 14, 16, 20, 22, 24, 26, 28, 30, 40, 50, 60, 70, 80, 90, 100, 120, 160, and 180 minutes. At 20 minutes, participants received a 0.03-U/kg infusion of regular human insulin (HumuLIN-R; Eli Lilly and Company) over 45 seconds to help assess the effect of insulin on glucose uptake. The test was stopped if the participant experienced severe neuroglycopenic symptoms. Any participant with screening hemoglobin levels below their sex-specific reference range but 10 g/dL or greater was given their 50-day course of iron tablets prior to being discharged. Glucose, insulin, and C-peptide were measured at Quest Diagnostics and the following methodology was used: spectrophotometry for glucose and immunoassay for insulin and C-peptide.
  - Body composition was measured subsequently by dual-energy x-ray absorptiometry (Lunar Prodigy version 8.50).

### MinMod Analysis

The MinMod Millennium software was used for this analysis [17]. This software is used to estimate critical indices of glucose-insulin dynamics during an FSIGT. The minimal model analysis allows quantitation of the ability of insulin to enhance glucose disposal by using rate constants characterizing glucose flux. The metabolic measures estimated in this fashion, for example, insulin sensitivity and glucose effectiveness, have been shown to correlate highly with the gold-standard euglycemic glucose clamp technique [15]. The glucose minimal model uses insulin observations to drive the glucose response and attempts to portray glucose dynamics in terms of 2 key parameters, glucose responsiveness and insulin sensitivity. The insulin minimal model uses glucose observations to drive the insulin response, and it attempts to portray insulin dynamics in terms of another set of key parameters, first-phase responsivity and second-phase responsivity. There are 4 reasonably distinct phases with this model: (1) a mixing phase, immediately following the glucose injection, and lasting for about 10 minutes, as glucose equilibrates in the circulation; (2) a phase of steady and almost constant decline as the concentration imbalance between circulating glucose and cellular glucose leads to glucose, largely, mediating its own disposal into the cell (minutes 12-20); (3) an extension of the glucose decline beyond that which would be anticipated based on “2” and due, primarily, to the action of exogenous interstitial insulin to prolong the disposal of glucose (minutes 22-50); and (4) a phase of recovery of glucose back to its basal value (minutes 60-180) [17].

### Indices of Insulin Resistance

The indices of insulin resistance were estimated on: (i) the fasting insulin and C-peptide levels; (ii) the glucose response to its own mediated action, and insulin infusion was calculated as the area under the curve (AUC) for glucose (AUC_glucose_) during the FSIGT: The AUC_glucose_ during 0 to 10 minutes represents the acute response to glucose load; the AUC_glucose_ during 12 to 20 minutes represents the ability for glucose to mediate its own disposal into the cells; the AUC_glucose_ during 22 to 50 minutes represents the glucose response to the action of exogenous interstitial insulin; and the AUC_glucose_ during 60 to 180 minutes is the phase of recovery of glucose back to its basal value; (iii) the insulin sensitivity (S_I_) and glucose effectiveness (S_G_): S_I_ and S_G_ were calculated with the minimal model software [17] (discussed earlier). S_I_ is defined as fractional glucose disappearance per insulin concentration unit. S_G_ is defined as the ability of glucose per se to promote its own disposal and inhibit hepatic glucose production in the absence of an incremental insulin effect; (iv) the homeostatic model assessment of insulin resistance (HOMA-IR): HOMA-IR correlates well hyperinsulinemic-euglycemic clamps and minimal model estimates of insulin resistance in prior studies [18, 19]; and (v) the homeostatic model assessment of insulin sensitivity (HOMA-SI) [18, 19].

### Indices of Pancreatic β-Cell Function (Insulin Secretary Function)

The indices of pancreatic β-cell function were estimated based on the following: (i) The first and second phase of insulin secretion: During the FSIGT, the first phase of insulin secretion or acute insulin response to glucose (AIRg) is represented by insulin being released after glucose injection, and was calculated using the AIRg calculation with the minimal model analysis [17] (discussed earlier) and the AUC of insulin and C-peptide concentration (AUC_insulin_ and AUC_Cpeptide_) during the first 10 minutes. The second phase of insulin secretion was calculated as the AUC for both parameters during 12 to 20 minutes after glucose bolus (prior to exogenous insulin administration); (ii) the HOMA of β-cell function (HOMA-β) is a static assessment of β-cell function using basal values of glucose and insulin or C-peptide [19] and was calculated based on fasting glucose, insulin, and C-peptide levels using the HOMA2 calculator [18]; (iii) D_I_ is expressed by calculating the product of insulin secretory capacity and insulin sensitivity [17] and is an indicator of β-cell function.

### Mass General Brigham Biobank Common Variant Associations

Common *FGFR1* variants (ie, SNVs) that have previously been linked to clinical phenotypes were identified via the GWAS catalog [20]. Using the MGBB portal, the associations of those common *FGFR1* variants were tested with already the curated clinical phenotype of T2D: 65 247 MGBB participants with genotyping data: rs9657190 rs881301, rs881299, rs7828172, rs6984358, rs66504003, rs62505473, rs60527016, rs59498392, rs57709857, rs4739558, rs4647906, rs4647903, rs4082204, rs3925, rs36061954, rs34036147, rs328301, rs3213849, rs2468964, rs2304000, rs222439, rs4733946, rs144330574, rs13317, rs13248631, rs11986274, rs10958704, and rs10101096.

### Statistical Analysis

Distributions of the outcomes and clinical characteristics were reported using mean and SD for normally distributed values and median and first and third quartile for nonnormally distributed values. The normality of data distribution was tested with a Shapiro-Wilks test. Statistical comparisons were performed with a *t* test and nonparametric Wilcoxon rank sum test for normally and nonnormally distributed values, respectively. Values that were missing (44/3242 time points) or were found to be more than above and below the mean ±3 × SDs (outliers) were excluded from the analysis. General linear model was applied to adjust the effect of *FGFR1* mutation carrier status for BMI and percentage of fat mass. Logarithmic transformation was applied for nonnormally distributed values by which the normality of all transformed variables was tested again with a Shapiro-Wilks test. *P* values less than .05 were considered statistically significant. For the common variant analysis, a Fisher exact test was used, and a statistical significance was defined by a *P* value with a cutoff of less than .012 (multiple testing correction for the identified variants).

## Results

### *FGFR1*m Patient Cases Show a Higher Percentage of Total Body Fat Compared to Controls (*FGFR1*c)

Nine *FGFR1*m patient cases and 27 noncarrier controls were enrolled and completed the study. The average age of the *FGFR1*m cases was 32 years, consistent with the young age at which individuals with IHH usually present at the clinic due to pubertal failure (Table 1). The average age of the *FGFR1*c controls was 47 years, which is in line with the fact that 46% of individuals enrolled in the MGBB are older than 60 years and only 24% of the MGBB participants are younger than 40 years [21]. Given that the incidence of diabetes and obesity increases with age, we ensured that despite the age difference between the 2 groups, none of the enrolled participants were diagnosed with prediabetes or diabetes, which was reflected by the absence of fasting hyperglycemia and no differences in their HbA_1c_ levels (see Table 1). In addition, the BMI was also not different between the 2 groups (mean BMI of 28.5 in the *FGFR1*m patient cases vs 26.1 in the *FGFR1*c controls; *P* = .233). All IHH participants were maintained on their appropriate sex steroid treatment at the time of the study. All 7 *FGFR1*m male participants were treated with either transdermal or intramuscular testosterone, and their testosterone levels did not differ from the testosterone levels of the controls (385 ng/dL vs 448 ng/dL; *P* = .558). Both female *FGFR1*m participants were treated with hormone therapy at the time of the study (one participant with an estradiol patch and progesterone and the second with a combined oral contraceptive pill). Dual-energy x-ray absorptiometry scan assessment showed that the *FGFR1*m patient cases demonstrated a higher percentage of total body fat (37.8%) compared to noncarrier controls (30.8%, *P* = .008; see Table 1). No differences were observed in the lean mass of participants in those 2 groups (see Table 1).

**Table 1.** Baseline characteristics and adiposity indices of enrolled participants

Clinical and laboratory characteristics	*FGFR1-*mutation carriers (N = 9) Mean (SD)	*FGFR1* noncarrier controls (N = 27) Mean (SD)	*P*
Age, y	32 (14)	47 (11)	.002
Fasting glucose, mg/dL	88 (6)	85 (8)	.304
HbA_1c_, %	5.4 (0.8)	5.2 (0.3)	.27
TSH*^a^*	1.21 (1.13-2.19)	1.54 (1.22-2.35)	.452
Prolactin*^a^*	6.5 (4.5-8.6)	5.35 (4.4-7.1)	.628
BMI*^a^*	29.5 (23.2-30.5)	25.4 (22.4-29)	.432
Percentage of total body fat	37.8 (6.8)	30.8 (5.7)	.008
Lean muscle mass, g*^a^*	50 383 (47 435-77 585)	51 372 (40 420-60 636)	.193

*FGFR1*-mutation carriers demonstrate a higher percentage of their total body fat compared to noncarrier controls, despite nondifferent BMI levels between the two groups.

Abbreviations: BMI, body mass index; *FGFR1*, fibroblast growth factor receptor 1; HbA_1c_, glycated hemoglobin A_1c_; TSH, thyrotropin.

^*a*^Reported as median (first and third quartile) due to nonnormal distribution of the data.

### *FGFR1*m Patient Cases Demonstrate Higher Insulin Resistance Compared to Controls (*FGFR1*c)

**Figure 1.**
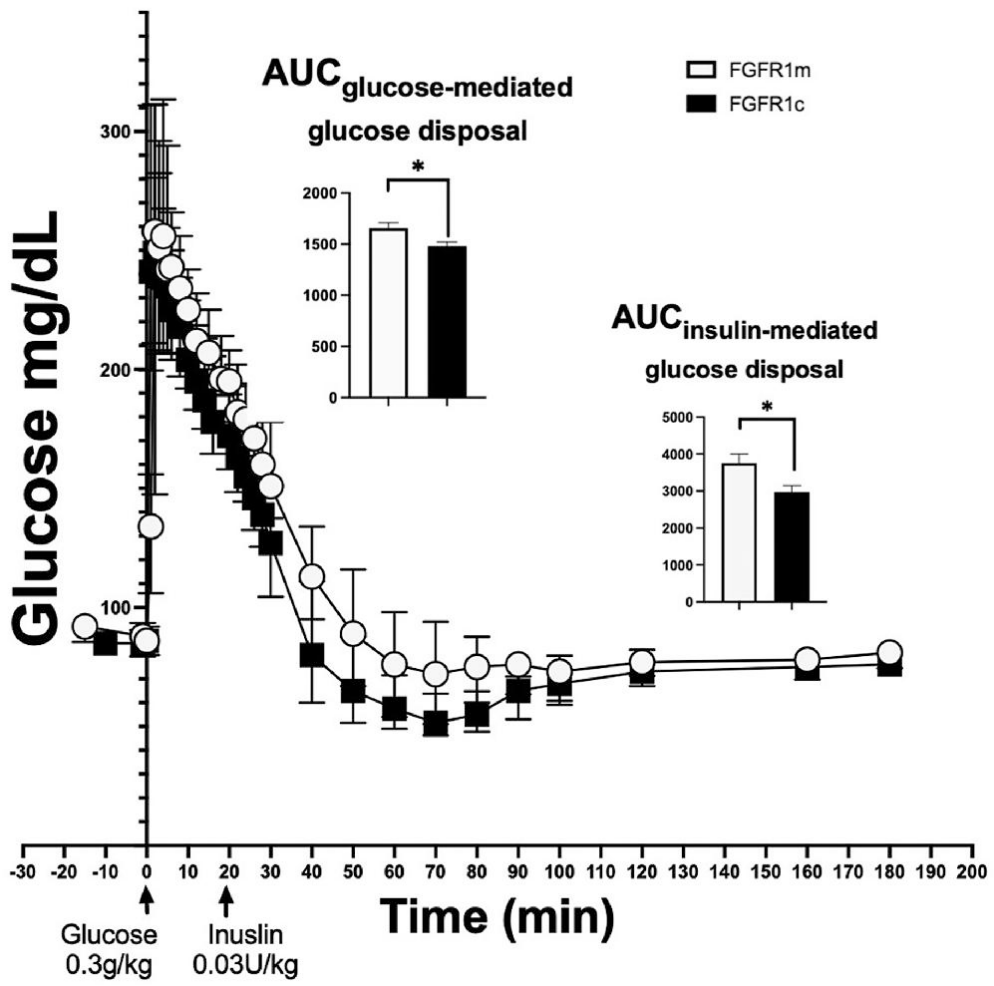
*FGFR1*m cases demonstrate higher glucose levels during the frequently sampled intravenous glucose tolerance test (FSIGT) phase of glucose-mediated and insulin-mediated glucose disposal compared to controls (*FGFR1*c). During the 4-hour FSIGT, baseline sampling for plasma glucose occurred at −10 and −1 minutes. These measurements were followed by administrating 0.3 g/kg bolus of glucose within 2 minutes. At 20 minutes, participants received a 0.03-U/kg infusion of regular human insulin over 45 seconds to enhance the insulin level to better help assess the effect of insulin on glucose uptake. *FGFR1*m cases (in white) demonstrated higher levels of glucose during the glucose-mediated (12-20 minutes) and insulin-mediated glucose disposal (22-50 minutes) compared to controls (ie, *FGFR1*c participants [in black]). *P less than .05; **P less than 001.

To investigate any differences in insulin sensitivity between the 2 groups, the glucose response to its own mediated action and to an insulin bolus were calculated using the AUC for glucose (AUC_glucose_) during the FSIGT. *FGFR1*m patient cases demonstrated higher AUC_glucose_ during minutes 12 to 20 of the study compared to noncarriers (Fig. 1). This phase represents their glucose-mediated glucose disposal. They also displayed a higher AUC_glucose_ during minutes 22 to 50, the phase that represents glucose's response to the action of exogenous insulin, compared to controls (see Fig. 1). While no differences in S_G_ were noted between the groups, S_I_, that is, the fractional glucose disappearance per insulin concentration unit, was lower in the *FGFR1*m patient cases compared to the healthy noncarrier controls (Table 2). These results were concordant with the higher fasting insulin and C-peptide levels, higher HOMA-IR, and the lower HOMA-SI calculated scores in the *FGFR1-*mutation carrier group compared to noncarrier controls (see Table 2), which remained significant after adjusting for BMI and percentage of total body fat (see Table 2).

**Table 2.** Indices of insulin resistance and secretary function in *FGFR1*-mutation carriers vs noncarrier controls

Indices of insulin resistance/secretion	*FGFR1*-mutation carriers (N = 9) Mean (SD)	Noncarrier controls (N = 27) Mean (SD)	*P*
Fasting glucose, md/dL	88 (6)	85 (8)	.304
Fasting insulin, μU/mL*^a,b^*	10.9 (4.8-12.3)	3.1 (1.8-5.5)	.002
Fasting C-peptide, ng/mL*^a,b^*	2.4 (2.2-2.9)	1.01 (0.71-1.91)	.001
HOMA-SI*^a,b^*	72 (63-163)	250 (137-270)	.002
HOMA-IR*^a,b^*	1.39 (0.61-1.58)	0.6 (0.4)	.002
HOMA-B*^a,b^*	132 (74-138)	70 (60-85)	.026
S_G_	0.022 (0.007)	0.019 (0.01)	.587
S_I_*^a^*	1.93 (1.46-2.54)	4.75 (2.26-7.87)	.022
D_I_	1530 (1240-2470)	1510 (891-2670)	.985
AIRg*^a^*	564 (342-880)	204 (150-585)	.0426

*FGFR1*-mutation carriers demonstrated higher fasting insulin and C-peptide levels compared to noncarrier controls. In addition, insulin sensitivity, as calculated by the S_I_ and HOMA-SI, was lower and insulin resistance, as shown by the HOMA-IR, was higher in the *FGFR1*-mutation carriers compared to noncarrier controls.

Abbreviations: AIRg, acute insulin response to glucose; D_I_, Disposition Index; *FGFR1*, fibroblast growth factor receptor 1; HOMA-B, homeostatic model assessment of β-cell function; HOMA-IR, homeostatic model assessment of insulin resistance; HOMA-SI, homeostatic model assessment of insulin sensitivity; S_G_, glucose effectiveness; S_I_, insulin sensitivity.

^*a*^Reported as median (first and third quartile) due to nonnormal distribution of the data.

^*b*^Remained significant after adjusting for body mass index and percentage of total body fat.

### *FGFR1*m Patient Cases Demonstrate Higher β-cell Function Compared to Controls (*FGFR1*c)

**Figure 2.**
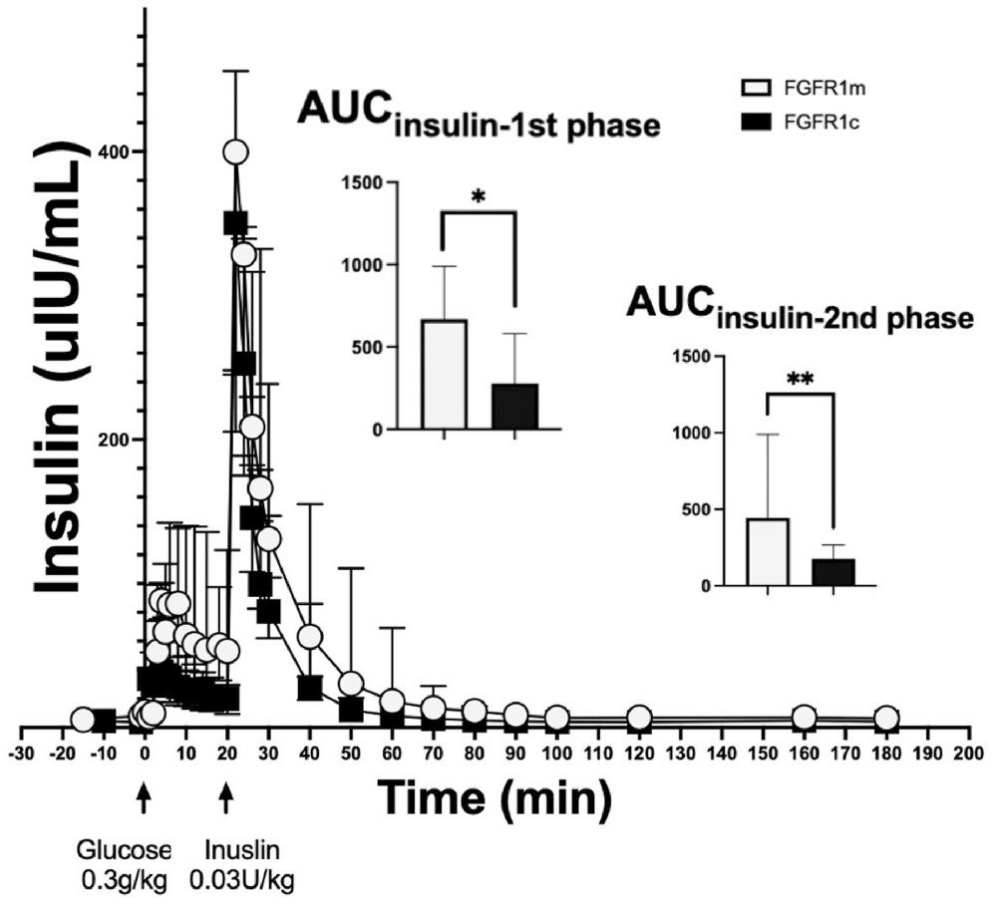
*Fgfr1*m cases demonstrate higher insulin response to glucose during the frequently sampled intravenous glucose tolerance test compared to controls (*FGFR1*c). The *FGFR1*m cases (shown in white) demonstrate higher insulin response to glucose compared to *FGFR1*c controls (shown in black), which was manifested during the first (0-10 minutes) and second phase (12-20) of the study. *P less than .05; **P less than 001.

**Figure 3.**
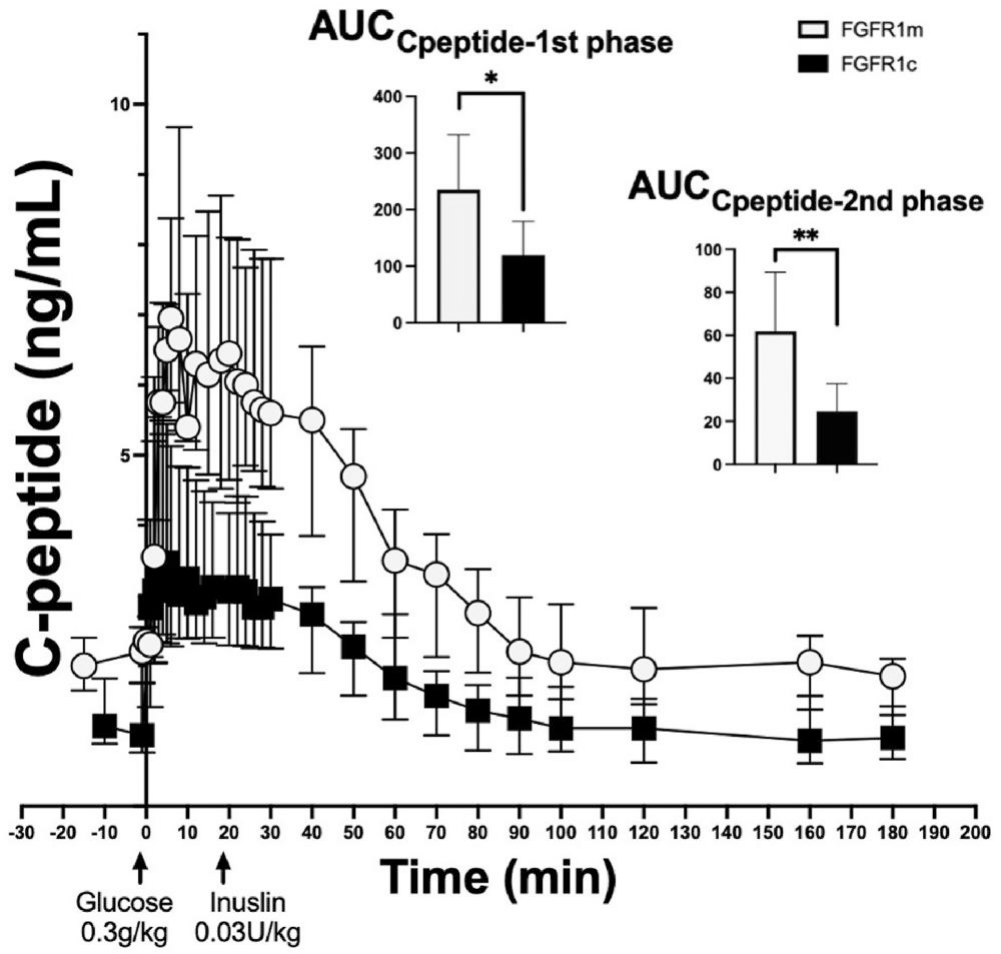
*FGFR1*m cases demonstrate higher C-peptide response to glucose during the frequently sampled intravenous glucose tolerance test compared to controls (*FGFR1*c). The *FGFR1*m cases (shown in white) demonstrate a higher C-peptide response to glucose compared to *FGFR1*c controls (shown in black), which was manifested during the first (0-10 minutes) and second phase (12-20) of the study. *P less than .05; **P less than 001.

During the FSIGT, *FGFR1*m patient cases demonstrated an increased AUC_insulin_ and AUC_Cpeptide_ for the total duration of the study, as well as during the first- and second-phase insulin secretion compared to healthy noncarriers (Figs. 2 and 3, respectively). These findings were consistent with the observed higher AIRg and the higher HOMA-β in the *FGFR1*m patient cases compared to controls (see Table 2). These findings suggest that the *FGFR1*m patient cases mounted a significantly higher β-cell response to maintain normal blood glucose levels. The D_I_, which is expressed by calculating the product of insulin secretory capacity and insulin sensitivity, was similar between the 2 groups. This finding reflected the compensatory higher β-cell function in the face of lower insulin sensitivity that the *FGFR1*m patient cases demonstrated compared to controls (see Table 2). Similar to the indices of insulin sensitivity/resistance, most of the indices of β-cell function remained significant when adjusting for BMI and total fat mass (see Table 2).

### *FGFR1* Common Alleles Are Associated With Differential Prevalence of Type 2 Diabetes in the Large Cohort of the Massachusetts General Brigham Biobank

**Figure 4.**
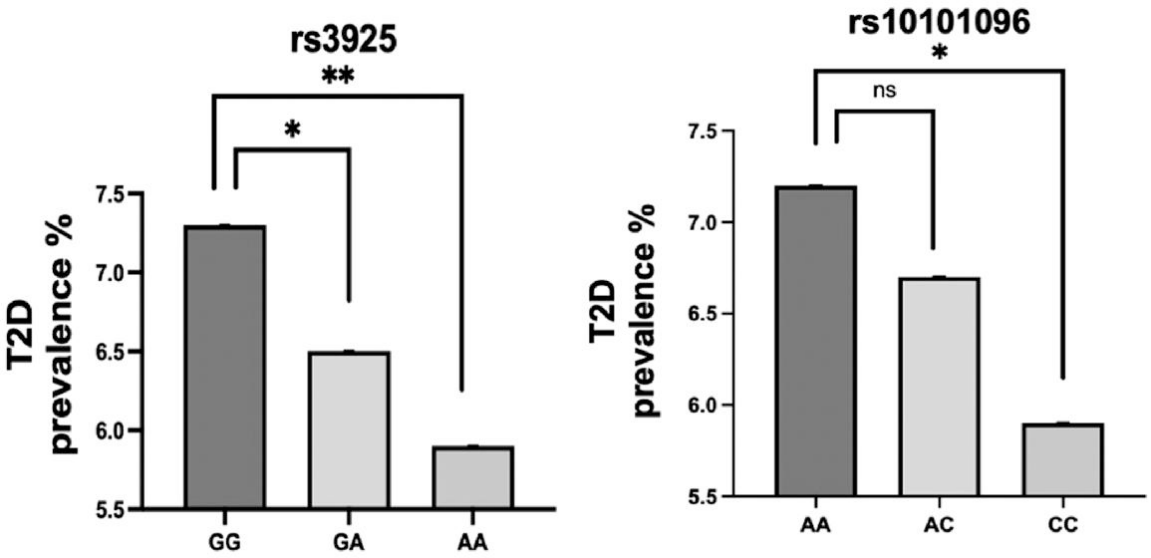
Common *FGFR1* alleles are linked to type 2 diabetes (T2D) prevalence in the Mass General Brigham Biobank (MGBB) population. MGBB individuals with the GG and GA genotypes in the rs3925 locus and individuals with the AA genotype in the rs10101096 locus demonstrated an increased risk of T2D compared to individuals with the AA and the CC genotype, respectively. *P less than .012 (0.05/number of variants tested); **P less than .001.

To further evaluate the role of FGFR1 signaling on glucose metabolism beyond the rare variant spectrum, we examined the association between *FGFR1* common variants and T2D in the MGBB. Among the consented participants in the MGBB with genomic data (N = 65 247), 6768 individuals were diagnosed with T2D based on curated phenotypic data (positive predictive value = 0.99). As shown in Fig. 4, the risk of T2D diagnosis increased with 2 common *FGFR1* genotypes: rs3925 and rs10101096. Specifically, for the genotype rs3925, T2D prevalence increased significantly for individuals with the homozygous GG alleles and the heterozygous GA alleles compared to the individuals carrying the homozygous the AA alleles. Similarly, the prevalence of T2D increased significantly for participants with the homozygous AA alleles at the rs10101096 locus compared to the carriers of the homozygous CC alleles.

## Discussion

Genotype-first approaches and targeted recall-by genotypes studies represent powerful tools in genomic medicine. Such approaches allow the early diagnosis of individuals with mutations in disease-causing genes and permit the discovery of novel genotype-phenotype associations. Using such an approach, we investigated whether rare *FGFR1* deleterious SNVs impaired glucose metabolism in humans. We show that participants with rare heterozygous deleterious *FGFR1* SNVs demonstrate higher insulin/C-peptide response to glucose and higher insulin resistance compared to noncarriers. Further, supported by the higher prevalence of T2D in individuals harboring common *FGFR1* risk alleles, this study now provides robust human genetic evidence of the critical role of FGFR1 signaling in glucose homeostasis.

The importance of impaired insulin release and insulin resistance in the pathogenesis of T2D is well known and has been evaluated in numerous prior studies [22-24]. Insulin sensitivity appears to decrease approximately 5 years prior to the development of T2D, while insulin secretion increases 3 to 5 years prior to the diagnosis, likely as a compensatory mechanism, to then decrease as individuals get closer to the development of T2D [22-25]. Compensatory insulin secretion by the pancreatic β cells initially maintains normal plasma glucose levels, but β-cell function progressively worsens over time [26]. As a result, increased insulin secretion and resistance are considered independent risk factors for T2D [22]. This is also true for high-risk patients, that is, patients already diagnosed with prediabetes, in whom a combination of decreased baseline insulin sensitivity and secretion appears to act additively to increase the risk for T2D development over time [27]. The hyperinsulinemia and lower insulin sensitivity observed in our study participants in the absence of overt hyperglycemia suggest that FGFR1 signaling may be temporally implicated in the early insulin hypersecretory phase and insulin resistance seen in initial stages of T2D pathophysiology. Despite the observed dysregulated metabolic indices, due to the study design wherein the presence of overt diabetes was considered an exclusion criterion, none of the *FGFR1*m patient cases carried a diagnosis of T2D. To further clarify the role of *FGFR1* variants in T2D within a population setting, we examined the association of common *FGFR1* variants with the prevalence of T2D within the MGBB. In line with prior reported studies [5, 6], common *FGFR1* genotypes were linked to T2D prevalence among MGBB participants. All common variant risk genotypes harbored noncoding possibly regulatory variants that mapped to the *FGFR1* gene locus. The precise mechanisms by which these genotypes alter the T2D risk remains unclear and will require further interrogation. Taken together, the results of this report strongly suggest that carriers of deleterious *FGFR1* SNVs and those possibly those harboring *FGFR1* common risk alleles should also be considered at higher risk of T2D and should be monitored for the development of the disease.

While BMI was not different between the 2 groups of this study, *FGFR1*m patient cases demonstrated a higher percentage of total body fat mass compared to controls. The differences in the fat distribution between the 2 groups could potentially explain the insulin resistance seen in the IHH *FGFR1*m participants, as abdominal obesity is often linked to ectopic fat deposition (eg, in muscle and liver) and may underlie the resistance to the effects of insulin on peripheral glucose and fatty acid utilization observed in patients with low insulin sensitivity, prediabetes, and diabetes [22, 28, 29]. However, β-cell function, insulin sensitivity, and insulin resistance remained significantly different in *FGFR1*m participants even after controlling for these adiposity indices. This suggests that the underlying defects in the FGFR1 signaling per se is likely to contribute to the glucose dysregulation observed in this study rather than their body composition differences. This assertion is also supported by prior studies showing that mice with dominant-negative loss of FGFR1 demonstrate abnormal β-cell differentiation, fasting, and nonfasting hyperglycemia (ie, diabetes-like phenotypes), despite being lean [1]. Possible alternate mechanisms that could explain the differences observed between the 2 groups may include altered thermoregulation and hypothalamic or peripheral regulation of browning of the white adipose tissue that is mediated via the FGFR1 signaling [30-32]. In addition, FGFR1 is highly expressed in the human adipose tissue, and specific knockout of FGFR1 eliminates the beneficial effects of FGF21, including weight loss and energy expenditure in obese rodent models [33], suggesting of an important role of FGFR1 expression in white adipose tissue. It is likely that the human carriers of deleterious FGFR1 variants may have defective signaling within the adipose tissue. Obtaining an adipose tissue biopsy in the enrolled participants and assessment of visceral adiposity measures should be addressed in future studies.

While the inactivation of the FGFR1 signaling pathway worsened insulin resistance in this study, prior studies have shown the beneficial effect of the activation of the FGFR1 signaling pathway on the metabolic health of animals and humans [2-4]. Hence a key biologic question arising from these observations relates to the identity of the specific FGF ligand(s) that may underlie the observed metabolic phenotype resulting from altered FGFR1 signaling. Among the multiple FGF ligands, several have been implicated in glucose regulation through different mechanisms: (i) Mice lacking fgf1 develop marked hyperglycemia and insulin resistance when challenged with a high-fat diet and in *ob/ob* and *db/db* mice or diet-induced obesity (DIO) models, peripheral delivery of a single dose of recombinant FGF1 can normalize blood glucose levels within hours, without inducing hypoglycemia, making Fgf1 a promising therapeutic agent of diabetes and insulin resistance [34, 35]; (ii) FGF2b may transform the nonendocrine human pancreatic cells into endocrine insulin-secreting cells [36]; (iii) FGF7 has been shown to enhance islet engraftment and improve metabolic control following islet transplantation in diabetic mice [37]; (iv) administration of FGF19 to obese diabetic mice leads to beneficial effects on metabolism [38]; and (v) exogenous FGF21 treatment of animal models reduces hyperglycemia by preventing islet destruction and improving glucose clearance and decreases their body weight and circulating lipids [39-42], while administration of FGF21 molecules in humans leads to a decrease in body weight, and an improvement of dyslipidemia [43-50].

Of the aforementioned FGF ligands, data from humans strongly suggest that FGF21 may be the primary driver of metabolic health relating to FGFR1 signaling. FGF21 is an endocrine FGF ligands that acts through the FGFR1-β-klotho complex and appears to have a crucial role in metabolic regulation. Specifically, higher serum FGF21 concentrations have been described in humans with obesity and diabetes, suggesting the potential presence of FGF21 resistance in addition to insulin resistance [51-54]. Further support for the role of FGF21 in improving metabolic health comes from studies that used agonistic FGFR1 receptor antibodies that mimic the action of FGF21. Those studies resulted in improvement of hyperglycemia, hyperinsulinemia, hyperlipidemia, and hepatosteatosis in obese diabetic mice [4], and significant weight loss in obese monkeys [3]. Further, in a recent clinical study, overweight human participants who received a single dose of a similar-acting antibody showed a transient weight reduction, improvement in cardiometabolic parameters, and reduction in carbohydrate intake [2]. Furthermore, FGF21 analogues have been studied both in animal and human models as therapeutics for obesity, T2D, hyperlipidemia, and nonalcoholic steatohepatitis [31, 44-47, 55-60]. The present study used an FSIGT method to assess glucose metabolism and hence potential incretin effects (eg, glucagon-like peptide-1 [GLP-1] axis) on glucose metabolism would be missed. While no direct relationship between FGFR1 signaling and the GLP-1 axis has been reported previously, GLP-1 receptor analogues appear to stimulate hepatic FGF21 production and inhibit gluconeogenesis [61]. This latter observation suggests that at least part of the FGF21's effects on glucose metabolism may be incretin mediated. In summary, while FGF21 was not directly studied in the present report, prior studies strongly implicated FGF21-FGFR1 signaling as the primary pathway for metabolic regulation in humans. The potential mechanisms for the role of FGF21-FGFR1 signaling in metabolic health include an improvement of β-cell function and insulin sensitivity, decreased glucagon release, increased thermogenesis due to central activation of the sympathetic nervous system, and induction of energy expenditure via brown fat activation [54, 62].

The intersection between genes implicated in IHH and their potential role in metabolic regulation is intriguing. FGFR1plays an important role in the IHH architecture. Mice homozygous for hypomorphic *Fgfr1* alleles display a reduction in hypothalamic GnRH [63], while expression of a dominant-negative FGF receptor in mouse GnRH neurons results in a decreased number of GnRH neurons in the forebrain and late pubertal onset [64]. Similar to mice, our group and others have shown that rare deleterious genetic *FGFR1* variants are a leading cause of IHH [65, 66]. Our recent analysis of next-generation sequencing data from a large cohort of IHH patients showed that 12% of them carry rare variants in *FGFR1*, demonstrating the major role of this signaling pathway in the genetic etiology of IHH [11]. The FGF21-FGFR1 signaling complex also includes β-klotho, an essential coreceptor that has also been implicated in IHH as well as metabolic phenotypes. Specifically, a broad spectrum of metabolic phenotypes, including obesity/overweight, fasting hyperglycemia, and insulin resistance, has previously been reported in IHH individuals harboring rare damaging heterozygous SNVs in *KLB*, the gene encoding for β-klotho [67], and rare digenic LoF variants in *FGFR1* and *KLB* have been reported in patients with severe insulin resistance [68]. Interestingly, while the binding site of β-klotho to FGFR1 is independent of the common site that β-klotho uses to bind to FGF19 and FGF21, it overlaps with the binding site for ligands of the FGF8 subfamily and prevents the formation of the FGF8-FGFR1 complexes [69]. Mutations in *FGF8* contribute to the genetic etiopathogenesis of IHH, and specific *FGFR1* mutations decrease the signaling of FGF21 and FGF8 by impairing the association of FGFR1 with β-klotho, leading to both IHH and metabolic phenotypes [67, 69-72]. In addition, central and peripheral administration of FGF21 induces GnRH neuronal growth and stimulates the release of GnRH from the hypothalamus [67]. Thus, the regulation of both the GnRH neuronal and pancreatic cell function by the FGF21-β-klotho-FGFR1 signaling pathway raises the hypothesis of shared regulatory genetic networks between GnRH and β-cell pancreatic development, with the FGF21-β-klotho-FGFR1 signaling pathway linking metabolism to reproduction.

This study's main strength is the utilization of a genotype-first approach that capitalizes on naturally occurring *FGFR1*-damaging variants to study the effect of FGFR1 signaling on human metabolic health. Heterozygous dominant-negative and recessive hypomorphic mutations in *FGFR1* have been implicated in causing dominant and recessive forms of Hartsfield syndrome, which is characterized by holoprosencephaly, ectrodactyly, cleft lip/palate, intellectual disability, and hypogonadotropic hypogonadism [73], and transgenic mice expressing a dominant-negative version of the *FGFR1* demonstrate overt diabetes [1]. Hence, future studies in humans with dominant-negative FGFR1 mutations may help validate the observations of this study. Similarly, metabolic studies in other FGFR1-related human diseases, such as Pfeiffer syndrome, a disease caused by *FGFR1* gain-of-function variants [73, 74], may also provide valuable information. In addition, hypogonadism, the main feature of IHH, has been previously linked to insulin resistance, with prior studies showing that repletion of hypogonadal individuals with sex steroids improves their metabolic health [75-77]. Thus, hypogonadism may represent a potential confounder contributing to the metabolic dysregulation observed in this study. However, all IHH participants were supplemented with sex steroids at the time of this study, suggesting that the metabolic abnormalities observed is unlikely to be related to their current sex steroid milieu. However, potential long-term metabolic sequalae stemming from pubertal hypogonadism, which could be contributing to the current observations, cannot be excluded. In addition, while *FGFR1*m participants demonstrated increased first- and second-phase insulin and C-peptide secretion, future cellular model studies are required to examine the direct effect of FGFR1 on islet cell function. Moreover, T2D is a polygenic disease and known to be regulated by common variants in multiple loci. While polygenic risk scores were not calculated for the enrolled participants, none of them were diagnosed with T2D or prediabetes at the time [78]. However, potential confounding of the study findings by polygenic genetic risk cannot be fully excluded. In conclusion, using a genotype-first approach, we showed that *FGFR1* human deleterious variants lead to insulin resistance and may increase the risk for T2D. Our findings provide direct human genetic evidence to support prior studies investigating the beneficial effect of the FGFR1 signaling pathway as a therapeutic target of diabetes and obesity in humans.

## Data Availability

Data and materials will be made available by the authors individually on request subject to the data-sharing plan and consent provided by the study participants.

## Abbreviations

AIRg: acute insulin response to glucose
AUC: area under the curve
BMI: body mass index
D_I_: Disposition Index
FGF: fibroblast growth factor
*FGFR1*: fibroblast growth factor receptor 1
*FGFR1*m: *FGFR1* mutation carriers
*FGFR1*c: noncarrier controls
FSIGT: frequently sampled intravenous glucose tolerance test
GLP-1: glucagon-like peptide-1
GnRH: gonadotropin-releasing hormone
HbA_1c_: glycated hemoglobin A_1c_
HOMA-B: homeostatic model assessment of β-cell function
HOMA-IR: homeostatic model assessment of insulin resistance
HOMA-SI: homeostatic model assessment of insulin sensitivity
g9 IHH: isolated hypogonadotropic hypogonadism
LoF: loss of function
MGBB: Massachusetts General Brigham Biobank
MGH: Massachusetts General Hospital
PTV: protein-truncating variant
S_G_: glucose effectiveness
S_I_: insulin sensitivity
SNV: single-nucleotide variation
T2D: type 2 diabetes
WES: whole-exome sequencing
